# The maternal microbiome influence on pregnancy success: breeding comparison of germ-free and conventionalized mice

**DOI:** 10.1080/19490976.2025.2609405

**Published:** 2026-01-07

**Authors:** Chudan Xu, Simona Antonacci, Francine Z. Marques

**Affiliations:** a Hypertension Research Laboratory, Department of Pharmacology, Biomedical Discovery Institute, Faculty of Medicine, Nursing and Health Sciences, Monash University, Melbourne, Australia; b Victorian Heart Institute, Monash University, Melbourne, Australia; c Baker Heart and Diabetes Institute, Melbourne, Australia

**Keywords:** Microbiome, germ-free, breeding, offspring, pregnancy, DOHaD, microbiota, intergenerational

## Abstract

Germ-free (GF) animals, which are entirely devoid of all microorganisms, are one of the most powerful tools for studying the role of the microbiome in a phenotype, moving the microbiome field from association to causation. They allow the introduction of specific microbes or microbial communities to interrogate the causality of microbiomes in protecting against or contributing to a phenotype. Here, we report critical and underappreciated challenges in using GF models to investigate the intergenerational effects of maternal diet and microbiota on offspring health. Using 57 GF and littermate conventionalized GF dams, we observed unexpectedly high maternal (odds ratio 11.5, *p* < 0.0001) and offspring (odds ratio 4.12, *p* < 0.0001) mortality in GF animals. Remarkably, GF dams had an extremely low pregnancy and parturition (*p*_microbiome_ < 0.0001) and a high incidence of cecal torsion (18.2%) compared to the conventionalized group, underscoring the indispensable role of the maternal microbiome in reproductive success and early development. Notably, even conventionalized GF mothers on high-fiber diets exhibited poor fertility, suggesting that microbial colonization timing and maternal microbial capacity to metabolize fiber are crucial. These findings not only reveal significant limitations in GF breeding protocols but also indicate that the maternal microbiota might influence offspring health far earlier than previously recognized, with implications for the developmental origins of health and disease research.

## Introduction

Germ-free (GF) mice are entirely devoid of all microorganisms, and they are one of the most powerful tools for studying the role of the microbiome in a phenotype and for moving the microbiome field from association to causation. They allow the introduction of specific microbes or microbial communities to interrogate the causality of microbiomes in protecting against or contributing to a phenotype.^1^^,^^2^ If GF mice are colonized with the fecal or intestinal microbiome of a non-GF mouse with a diverse and largely undefined microbiome, they are referred to as ‘conventionalized’ or ‘colonized’ mice.^2^^,^^3^ These offer the best control for GF experiments, as they control for the genetic background and environmental conditions of GF mice. Additionally, GF mice can be manipulated to become humanized gnotobiotic mice via human-derived fecal microbiota transplantation (FMT). These are valuable tools for investigating how patient-derived microbiomes play a role in pathophysiological conditions.^4^ Furthermore, GF models provide a valuable tool for investigating drug‒microbiome interactions and optimizing therapeutic strategies. For example, pharmacomicrobiomics is an emerging field investigating how the microbiome metabolizes drugs and influences drug efficacy.^5^ While antibiotics are known to affect the gut microbiome, numerous non-antibiotic drugs, including proton pump inhibitors, metformin, and angiotensin-converting enzyme inhibitors, also modulate microbial composition and function.^5^ This interaction is bidirectional, as these drugs alter the microbiome while microbial communities, in turn, influence drug metabolism, efficacy, and safety.^5^ Nonetheless, the use of GF mice remains limited owing to high costs, the technical challenges associated with maintaining GF status, and the complexity of any manipulation, particularly surgical, which restricts their widespread adoption in research.

Maternal physiology and lifestyle during pregnancy are crucial in shaping offspring health and influencing disease risk into adulthood.^6^ While the concept of the developmental origins of health and disease (DOHaD) is well accepted, there has been a growing interest in understanding whether the gut microbiome is involved. Findings from the past decade suggest that the microbiome mediates at least part of these observed intergenerational effects, many of which are proposed to occur *in utero* through epigenetic mechanisms.^7^ For example, a maternal high-fiber diet protected offspring from allergic airway disease through *in utero* epigenetic imprinting in the lungs and immune cells, independent of maternal microbiota transfer.^8^ Several studies also showed that the maternal microbiota shaped the development of the offspring's innate immune system *in utero.*^9^ Although it remains unclear whether epigenetic changes occur as early as *in utero*, DNA methylation in the intestinal stem cells from birth to weaning had a lasting effect on barrier integrity.^10^ This is particularly relevant, as DNA methylation is strongly influenced by butyrate, a microbial metabolite produced from fiber fermentation, which, in newborns, relies on maternal fiber intake and the maternal microbiome during breastfeeding. Similarly, maternal diet influenced the offspring's predisposition to cardiovascular disease, with effects lasting into adulthood.^11^ In an angiotensin II-induced hypertension model, offspring of high-fiber-fed mothers exhibited reduced histone H3 acetylation at the natriuretic peptide A (*Nppa*) promoter, highlighting the intergenerational cardioprotective effects of maternal diet.^11^ However, whether these intergenerational effects occur *in utero* or are driven by the maternal microbiome acquired at birth remains debatable.

There are different approaches to examining and disentangling these effects: (1) breeding under GF conditions; (2) cesarean delivery with cross-fostering; (3) antibiotic treatment of dams or offspring; and (4) cohousing offspring to standardize their microbiome. Breeding under GF conditions is considered the gold standard method for evaluating the roles of the microbiome, while other methods have limitations. For example, cross-fostering is the exchange of newly born pups to non-birth mothers who have recently had pups or are ready to nurse.^12^ Caesarean delivery is often used to precisely time this exchange and prevent maternal microbiome transmission during natural birth. However, this method requires precise time mating and skilled surgical and animal handling. The success rate of cross-fostering was claimed to be 22%, excluding the C-section failure rate.^13^ Importantly, the procedure can induce stress in foster mothers, which can sometimes lead to pup neglect or cannibalism. Antibiotic treatments usually target a broad spectrum of bacteria and can potentially result in off-target effects and incomplete or inconsistent microbial depletion.^14^ Additionally, prolonged or repeated use may develop antibiotic resistance.^15^ Although a non-absorptive antibiotic cocktail has recently been developed and applied in a mouse study to target the intestinal flora specifically,^16^^,^^17^ most antibiotics are systemically absorbed and can produce confounding results by affecting host physiology beyond the gut microbiota. Cohousing is the standard method used to promote horizontal transmission of microbiota between different individuals, with the aim of normalizing the gut microbiota composition.^18^^,^^19^ This approach helps researchers pinpoint whether an observed phenotype is due to *in utero* developmental plasticity or early-life exposure to the maternal microbiome. However, this method is relatively slow, typically requiring a minimum of 28 d to standardize the microbiome.^20^ Furthermore, the microbiome homogenization can be only partial^21^ or asymmetrical between two mouse populations.^20^ This approach also requires time-mate breeding, but it offers a higher survival rate and more flexibility in birth timing compared to cross-fostering. On the other hand, conventionalizing GF animals requires minimal interventions, allowing them to serve as a colonized group while littermate GF animals act as controls. This approach should be particularly valuable for determining whether the maternal microbiome drives intergenerational effects.

The notion that a diet enriched in fiber lowers blood pressure and improves cardiometabolic health has received substantial experimental support across the epidemiological, clinical trials, and discovery science fields.^22^ Our team^23-26^ and others have consistently shown that dietary fiber intake is a strong modifiable factor shaping the gut microbiota in both mice^27-29^ and humans,^30-33^ primarily through microbial fiber fermentation and the production of microbial metabolites called short-chain fatty acids (SCFAs). Thus, we initially aimed to breed GF and littermate conventionalized GF mice on different diets to investigate the impact of the maternal diet and gut microbiome on offspring health, and to determine whether these effects were driven *in utero* or by the maternal microbiome acquired at birth. However, we came across unexpected breeding challenges using GF mice in this intergenerational study. Here, we report some challenges and their implications for breeding animals under GF conditions. We hope these valuable lessons can help guide others who seek to conduct similar studies using GF models.

## Methods

### Animal model and experimental intervention

**Figure 1.**
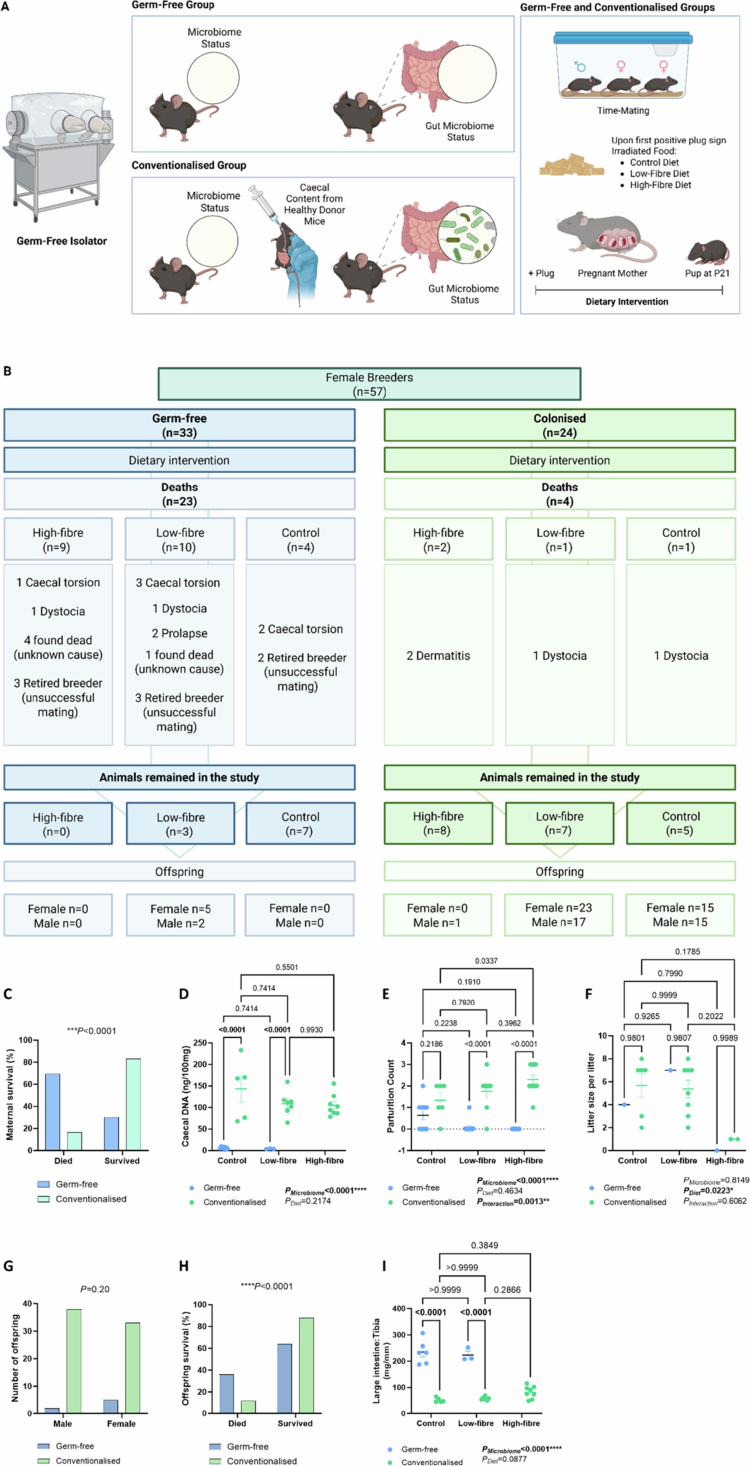
Intergenerational studies using germ-free mice. (A) Schematic representation of the experimental design. (B) Complications and mortality rates for germ-free and conventionalized female mice housed in a germ-free environment, along with the number of offspring produced by each group that survived until adulthood. (C) The percentage of maternal survival rate between the germ-free and conventionalized groups. (D) DNA quantification of the cecal contents from the dams (ng/100 mg). (E) Parturition count and (F)litter size per litter per dam at birth throughout the breeding study. (G) Number of offspring who survived until 12 weeks of age by sex. (H) The percentage of offspring survival rate between germ-free and conventionalized dams. (I) Large intestine-to-tibia ratio (mg/mm) of dams. Legends: Chi-square (C, G–H) and two-way ANOVA (D–F, I; all values are expressed as the mean ± SEM) tests were performed. Litter size was calculated as the number of pups born per litter per dam at birth. Panels A–B were created in BioRender. Panels C–I with GraphPad Prism.

Animal experiments were conducted in the Walter and Eliza Hall Institute Germ-free Unit with the approval of Monash University Animal Ethics Committees (38263) in compliance with National Medical and Health Research Council guidelines. Briefly, C57BL/6J female dams and male studs were born and raised in a GF environment. Nulliparous female mice between 7 and 31 weeks of age who had never been pregnant or given birth previously were randomized into the GF or conventionalized groups. For the latter, mice were introduced to a regular gut microbiota by oral gavage of a cecal slurry from healthy donors (Figure 1A; details described below). Both the GF and the conventionalized groups had the same housing conditions and breeding scheme to minimize confounding factors. The animals were time-mated and housed in flexible-film isolators in open-top cages. When a positive plug was observed, the studs were removed. Pups were weaned at day 21 (P21).

### Cecal slurry for oral gavage

The cecal contents from 10 adult female C57BL/6J mice bred under specific pathogen-free (SPF) conditions were collected and processed according to Choo and Rogers^34^ with modifications. Briefly, donor mice were humanely sacrificed via carbon dioxide (CO_2_) inhalation, and the ceca were collected into sterile, prereduced (with 0.05% L-cystine hydrochloride monohydrate) 15% glycerol solution in PBS (pH 7.4). Cecum tissues were immediately transferred to an anaerobic chamber (Whitley A35 Workstation), and the cecal contents were pulled together, weighed, homogenized by filtering through a 70 µm cell strainer, and diluted with a sterile, prereduced 30% glycerol solution in PBS (weight/volume ratio of 1:4). The suspension was mixed by vortexing, aliquoted and stored at −80 °C. The mice assigned to the conventionalized group received 200 μl of the suspension—pre-diluted 1:1 with reduced and sterile PBS upon thawing—via oral gavage once weekly for four consecutive weeks. The conventionalized group received the weekly cecal slurry via oral gavage and was closely monitored during and after the procedure for any distress and/or adverse signs. Mice were monitored for their welfare before proceeding with mating after four weeks.

### Dietary interventions

Once a positive plug was confirmed, female mice remained in the same cage and were randomized to one of three different irradiated diets (standard, high-fiber or low-fiber) for the remainder of their pregnancy and lactation periods. All the diets were supplied by specialty feeds (cat# SF19-211, SF23-112, SF23-113) (Table 1), which differed only in their fiber content or type (0% for low-fiber diet, 5.15% insoluble fiber in the control diet and high-fiber diet, having all the carbohydrates replaced with resistant starches, a highly fermentable type of fiber), and were formulated on the same AIN93G background, matching the content of carbohydrates, lipids and proteins.

**Table 1.** Mouse diet composition.

Specialty feeds code	SF19-211	SF23-112	SF23-113
	**Modified AIN93G**	**Modified SF09-028**	**Modified SF11-025**
	Modified AIN93G control diet	Low fiber, low starch diet	All CHO gel crisp starch, high-fiber diet
**Carbohydrates/fiber (69.7%)**			
**Digestible carbohydrates**	**23.20%**	**69.53%**	**0%**
Sucrose	10.00%	–	–
Dextrose monohydrate	–	69.53%	–
Dextrinized starch (Gemspray 17DE)	13.20%	–	–
**Starch**	**41.28%**	**0%**	**64.53%**
Starch	41.28%	–	–
Gel crisp (crisp film) (resistant) starch	–	–	64.53%
**Insoluble fiber**	**5.15%**	**0.15%**	**5.15%**
Cellulose (150)	5.00%	–	5.00%
Rat and mouse pellets (prescription)	0.15%	0.15%	0.15%
**Proteins (20.3%)**			
Casein (acid)	20.00%	20.00%	20.00%
L-Methionine	0.30%	0.30%	0.30%
**Lipids (7%)**			
Canola oil	7.00%	7.00%	7.00%
**Micronutrients/minerals/additives (3%)**			
Fine calcium carbonate	1.31%	1.31%	1.31%
Salt (fine sodium chloride)	0.26%	0.26%	0.26%
Potassium dihydrogen phosphate	0.69%	0.69%	0.69%
Potassium sulfate	0.16%	0.16%	0.16%
Potassium citrate	0.25%	0.25%	0.25%
Magnesium oxide	0.15%	0.10%	0.10%
Choline chloride 75% w/w	0.25%	0.25%	0.25%
Green food colour (E133 + E102)	0.002%	–	–

Note: All the diets were irradiated for germ-free conditions and supplied by specialty feeds.

## Endpoints

Female mice that were not found to be pregnant were re-mated a maximum of six times, in accordance with welfare requirements from the animal ethics committee. In the case of a successful pregnancy, the same dam was allowed to give birth to up to three litters. At the endpoint, the animals were humanely euthanized by CO_2_ induction, followed by tissue weighing and collection. Some animals presented with cecal torsion. This finding was identified post-mortem based on gross pathological signs. The animals were either euthanized due to clinical symptoms or found dead. Necropsy was performed on animals that died unexpectedly by experienced animal technicians. The main characteristics included a bloated abdomen, enlarged cecum size and/or twisted cecum.

## Mouse cecal DNA extraction and measurement

DNA extraction of cecal contents was performed using the DNeasy PowerSoil DNA isolation kit (Qiagen). The DNA samples were quantified using a Nanodrop (Thermo Fisher Scientific).

## Statistical analyses

GraphPad Prism (version 10.1.2) was used to plot and analyze the data. Differences in the percentage of maternal and offspring survival and the sex of the offspring were assessed using the chi-square test. A two-way ANOVA was performed to determine the effects of the microbiome and the dietary intervention on parturition count per dam, the litter size per dam, and the large intestine-to-tibia ratio. Where data permitted to fit a full model, interactions between diet and the microbiome were included. In cases where this was not feasible, only the main effects of either diet or the microbiome were analyzed. For litter size analysis, litter size was calculated as the number of pups born per litter per dam at birth, considering that most dams produced a single litter [conventionalized and GF groups, *n* = (8 + 2)/14, 71.4%], and only a few (*n* = 2/conventionalized groups—control and low-fiber diets) produced two litters; thus, each litter was considered as an independent reproductive event. Significance was set as *p* < 0.05.

## Results and discussion

To investigate whether the intergenerational effects of maternal diet are primed *in utero* or driven by the maternal microbiome acquired at birth, we conducted a breeding experiment in a GF environment for more than five months. We bred 57 dams, of which 33 were GF and 24 were colonized. DNA quantification of the cecal contents confirmed the success of oral gavage and verified that the germ-free animals remained devoid of microbial colonization in the large intestine (Figure 1D, *p*_microbiome_ < 0.0001). GF dams had a significantly higher death rate than conventionalized GF mice (Figure 1B,C, odds ratio 11.5, *p* < 0.0001). Not surprisingly, the introduction of a healthy gut microbiome significantly alleviated the complications and sudden deaths observed in GF animals, which is consistent with the long-established understanding that the gut microbiota supports host physiological stability and resilience.^35^^,^^36^ What was unexpected, however, was that only one successful litter of seven pups came from a GF mother (Figure 1B), highlighting the extremely low pregnancy and birth rates of GF dams. This finding was also reflected in the parturition count per animal, indicating that the absence of the microbiome was the primary factor in lower successful reproduction, which explained 52.69% of the total variation in the number of parturitions (Figure 1E, *p*_microbiome_ < 0.0001). Interestingly, conventionalized dams fed on a high-fiber diet had a higher parturition count than those on a control diet (Figure 1E, *p* = 0.0337). Furthermore, maternal dietary fiber intake had an effect on the litter size at birth (Figure 1F, *p*_diet_ = 0.0223), independent of individual dam variability or the microbiome, as each dam contributed one to two litters to the study. However, further validation is needed due to the unbalanced sample size between the germ-free and conventionalized groups in this study.

Out of 92 pups produced in this study, we did not observe any differences between the number of female and male offspring between GF and conventionalized GF mice (Figure 1G, *p* = 0.21). Seventy-eight (84.8%) offspring survived until 12 weeks of age as an endpoint (Figure 1B), of which 91% were part of a conventionalized litter, whereas only 9% came from GF parents (Figure 1H, odds ratio 4.12, *p* < 0.0001). We investigated whether GF mice had enlarged ceca, which reduce the abdominal space and thereby limit reproductive capacity.^37^ We assessed the size of the large intestine, in which the cecum represents the major anatomical component and principal site of fiber fermentation in mice, normalized to tibia length for comparison across groups. We found that the absence of the microbiome was the main factor that led to enlarged ceca in GF animals compared to the conventionalized group (Figure 1I, *p*_microbiome_ < 0.0001), suggesting that an enlarged cecum may lead to reduced reproductive capacity.

Remarkably, even conventionalized mothers on a high-fiber diet showed poor fertility, contributing only 4.9% of the total offspring in the colonized group, with only one pup surviving until the endpoint (Figure 1B). In contrast, conventionalized mothers on low-fiber and control diets accounted for 53.1% and 42% of the offspring, respectively. These findings suggest that conventionalized mothers, born in a GF environment, had a greater impact on their ability to utilize fiber and that the female microbiome may be critical in their fertility and/or fetal development, potentially influencing it much earlier than previously thought. Supporting this hypothesis, a recent study demonstrated that the maternal gut microbiota influences offspring stem cell characteristics.^38^ Specifically, exposure to *Akkermansia muciniphila* in mothers had beneficial effects on neuronal and intestinal stem cells in their offspring.^38^

Another major issue we encountered was the high incidence of cecal torsion or volvulus in our GF mouse colony, with an estimated 18.2% of cases confirmed post-mortem in GF female mice. It is well documented that GF rodents develop an enlarged cecum, a characteristic first reported in GF neonatal rats.^39^ In our study, we found that the absence of a microbiome was responsible for 44.25% of the large intestinal weight, significantly increasing it relative to conventionalized GF mice, independent of the diet (Figure 1I, *p* < 0.0001). This anatomical feature increases the risk of digestive tract twisting, leading to cecal torsion or volvulus, which can result in intestinal obstruction, compromized blood supply, and death.^40^^,^^41^ This outcome was expected in the GF environment, as the absence of the gut microbiota and its associated enzymes and by-products leads to osmolarity changes in the intestinal lumen. Microbial enzymes degrade many macromolecules, one example being mucopolysaccharides, which are long chains of sugar molecules. Without the gut microbiota, these molecules retain water, contributing to cecal dilation.^42^ Our data suggest that the combination of GF status and high-fiber intake may exacerbate cecal distension and torsion risk, contributing to a higher mortality and, potentially, poor reproductive outcomes. Interestingly, the mouse genetic background also influences the extent of cecal enlargement, with the C57BL/6JZtm (B6J) strain exhibiting the most pronounced effect among the strains compared, including NMRI/MaxZtm (NMRI), Balb/cJZtm (BALBc), 3H/HeOuJZtm (C3H), and C57BL/6NRjZtm (B6N).^42^ Nevertheless, the unexpectedly high incidence rate of cecal torsion required adjustments, including an increased demand for sample sizes, increased technical maintenance, a greater supply of irradiated food, and prolonged experimental time.

Consistent with the DOHaD framework, maternal nutrition is a well-established determinant of early-life development, with the maternal gut microbiota playing a critical role in this process. Beyond aiding digestion, maternal microbes contribute essential vitamins and amino acids, detoxify xenobiotics, and break down otherwise indigestible dietary components such as fiber.^43^ A healthy maternal microbiota is, therefore, essential, as it facilitates the maternofetal molecular transfer and directly influences foetal development.^43^ Even before birth, metabolites derived from the maternal gut microbiota penetrate nearly every organ system *in utero* by crossing the placental barrier.^44^ For instance, microbial metabolite vitamin K is crucial for normal blood clotting,^45^ microbial-produced folate (vitamin B9),^43^ and secondary bile acids^44^ are known to support embryonic and foetal development. These findings highlight the profound impact of the maternal microbiome not only on foetal development but potentially even earlier. This may partly explain the extremely low birth rates observed in GF mice, reinforcing the indispensable role of maternal microbes in reproductive success.

This study has several limitations that warrant consideration. First, we observed an unexpectedly high incidence of cecal torsion in GF mice (18%), which may have contributed to the poor reproductive outcomes and elevated mortality in this cohort. While cecal torsion has been reported in GF colonies, our rate exceeds historical baselines and may reflect the combined impact of GF status and a high-fiber dietary intervention, a combination not widely studied. The underlying mechanism, however, remains to be elucidated. Second, although we controlled for age, baseline parity, housing and procedural variables, the physiological mechanisms underlying poor reproductive success in GF dams remain unclear. Factors such as impaired fiber fermentation, absence of microbial-derived metabolites (e.g., SCFAs), altered immune signaling, and maternal care deficits may all contribute but were beyond the scope of this study. Furthermore, our study was not designed to dissect mechanistic pathways such as gut–immune–reproductive axis signaling or local versus systemic SCFA effects. These questions require targeted experimental designs, including tissue-specific metabolite profiling, immune cell tracking, and potentially transgenic or chimeric models—approaches that extend beyond the scope of this breeding-focused investigation. We highlight these limitations to encourage further research into the complex interactions between microbiome, dietary fiber, and reproductive physiology in intergenerational studies.

In conclusion, breeding GF animals in GF conditions is challenging for intergenerational studies where dietary interventions, particularly diets high in fiber, are used. The complete absence of the gut microbiota in GF mice leads to significant challenges, including lower pregnancy and birth rates and maternal and offspring survival, underscoring the importance of a healthy maternal microbiome. These effects are potentially primed well before the newborn acquires its microbiota after birth.

## Data Availability

The data that support the findings of this study are openly available in Zenodo at https://doi.org/10.5281/zenodo.15628482.

## References

[1] Schaedler RW, Dubs R, Costello R. Association of germfree mice with bacteria isolated from normal mice. J Exp Med. 1965;122(1):77–82. doi: 10.1084/jem.122.1.77. 14325475 PMC2138033

[2] Gordon HA, Pesti L. The gnotobiotic animal as a tool in the study of host microbial relationships. Bacteriol Rev. 1971;35(4):390–429. doi: 10.1128/br.35.4.390-429.1971. 4945725 PMC378408

[3] Souza DG, Vieira AlT, Soares AC, Pinho V, Nicoli JR, Vieira LQ, Teixeira MM. The essential role of the intestinal microbiota in facilitating acute inflammatory responses. J Immunol. 2004;173(6):4137–4146. doi: 10.4049/jimmunol.173.6.4137. 15356164

[4] Park JC, Im S-H. Of men in mice: the development and application of a humanized gnotobiotic mouse model for microbiome therapeutics. Exp Mol Med. 2020;52(9):1383–1396. doi: 10.1038/s12276-020-0473-2. 32908211 PMC8080820

[5] Weersma RK, Zhernakova A, Fu J. Interaction between drugs and the gut microbiome. Gut. 2020;69(8):1510–1519. doi: 10.1136/gutjnl-2019-320204. 32409589 PMC7398478

[6] Fleming TP, Watkins AJ, Velazquez MA, Mathers JC, Prentice AM, Stephenson J, Barker M, Saffery R, Yajnik CS, Eckert JJ, et al. Origins of lifetime health around the time of conception: causes and consequences. Lancet. 2018;391(10132):1842–1852. doi: 10.1016/S0140-6736(18)30312-X. 29673874 PMC5975952

[7] Yang C, Snelson M, El-Osta A, Marques FZ. Parental diet and offspring health: a role for the gut microbiome via epigenetics. Nat Rev Gastroenterol Hepatol. 2025;22:755–772. doi: 10.1038/s41575-025-01106-3. 40903589

[8] Thorburn AN, McKenzie CI, Shen S, Stanley D, Macia L, Mason LJ, Roberts LK, Wong CHY, Shim R, Robert R, et al. Evidence that asthma is a developmental origin disease influenced by maternal diet and bacterial metabolites. Nat Commun. 2015;6:7320. doi: 10.1038/ncomms8320. 26102221

[9] Kalbermatter C, Fernandez Trigo N, Christensen S, Ganal-Vonarburg SC. Maternal microbiota, early life colonization and breast milk drive immune development in the newborn. Front Immunol. 2021;12, 10.3389/fimmu.2021.683022. PMC8158941 34054875

[10] Yu D-H, Gadkari M, Zhou Q, Yu S, Gao N, Guan Y, Schady D, Roshan TN, Chen M, Laritsky E, et al. Postnatal epigenetic regulation of intestinal stem cells requires DNA methylation and is guided by the microbiome. Genome Biol. 2015;16(1):211. doi: 10.1186/s13059-015-0763-5. 26420038 PMC4589031

[11] Jama HA, Dona MSI, Dinakis E, Nakai M, Paterson MR, Shihata WA, Krstevski C, Cohen CD, Weeks KL, Farrugia GE, et al. Maternal diet and gut microbiota influence predisposition to cardiovascular disease in offspring. Circ Res. 2024;135(4):537–539. doi: 10.1161/CIRCRESAHA.124.324614. 39016011

[12] Daft JG, Ptacek T, Kumar R, Morrow C, Lorenz RG. Cross-fostering immediately after birth induces a permanent microbiota shift that is shaped by the nursing mother. Microbiome. 2015;3:17. doi: 10.1186/s40168-015-0080-y. 25969735 PMC4427954

[13] Yeom SC, Yu SA, Choi EY, Lee BC, Lee WJ. Prevalence of helicobacter hepaticus, murine norovirus, and pneumocystis carinii and eradication efficacy of cross-fostering in genetically engineered mice. Exp Anim. 2009;58(5):497–504. doi: 10.1538/expanim.58.497. 19897933

[14] Kennedy EA, King KY, Baldridge MT. Mouse microbiota models: comparing germ-free mice and antibiotics treatment as tools for modifying gut bacteria. Front Physiol. 2018;9:1534. doi: 10.3389/fphys.2018.01534. 30429801 PMC6220354

[15] Xu L, Surathu A, Raplee I, Chockalingam A, Stewart S, Walker L, Sacks L, Patel V, Li Z, Rouse R. The effect of antibiotics on the gut microbiome: a metagenomics analysis of microbial shift and gut antibiotic resistance in antibiotic treated mice. BMC Genom. 2020;21(1):263. doi: 10.1186/s12864-020-6665-2. PMC7106814 32228448

[16] Nevado R, Forcén R, Layunta E, Murillo MD, Grasa L. Neomycin and bacitracin reduce the intestinal permeability in mice and increase the expression of some tight-junction proteins. Rev Esp Enferm Dig. 2015;107(11):672–676. doi: 10.17235/reed.2015.3868/2015. 26541656

[17] Argaw-Denboba A, Schmidt TSB, Di Giacomo M, Ranjan B, Devendran S, Mastrorilli E, Lloyd CT, Pugliese D, Paribeni V, Dabin J, et al. Paternal microbiome perturbations impact offspring fitness. Nature. 2024;629(8012):652–659. doi: 10.1038/s41586-024-07336-w. 38693261 PMC11096121

[18] Lipinski JH, Zhou X, Gurczynski SJ, Erb-Downward JR, Dickson RP, Huffnagle GB, Moore BB, O’Dwyer DN, Raffatellu M. Cage environment regulates gut microbiota independent of toll-like receptors. Infect Immun. 2021;89(9):e0018721. doi: 10.1128/IAI.00187-21. 33941577 PMC8370678

[19] Garrett WS, Lord GM, Punit S, Lugo-Villarino G, Mazmanian SK, Ito S, Glickman JN, Glimcher LH. Communicable ulcerative colitis induced by T-bet deficiency in the innate immune system. Cell. 2007;131(1):33–45. doi: 10.1016/j.cell.2007.08.017. 17923086 PMC2169385

[20] Caruso R, Ono M, Bunker ME, Núñez G, Inohara N. Dynamic and asymmetric changes of the microbial communities after cohousing in laboratory mice. Cell Rep. 2019;27(11):3401–3412 e3. doi: 10.1016/j.celrep.2019.05.042. 31189120 PMC6690056

[21] Robertson SJ, Lemire P, Maughan H, Goethel A, Turpin W, Bedrani L, Guttman DS, Croitoru K, Girardin SE, Philpott DJ. Comparison of co-housing and littermate methods for microbiota standardization in mouse models. Cell Rep. 2019;27(6):1910–1919 e2. doi: 10.1016/j.celrep.2019.04.023. 31067473

[22] Xu C, Marques FZ. How dietary fibre, acting via the gut microbiome, lowers blood pressure. Curr Hypertens Rep. 2022;24:509–521. doi: 10.1007/s11906-022-01216-2. 35838884 PMC9568477

[23] Marques FZ, Nelson E, Chu P-Y, Horlock D, Fiedler A, Ziemann M, Tan JK, Kuruppu S, Rajapakse NW, El-Osta A, et al. High-fiber diet and acetate supplementation change the gut microbiota and prevent the development of hypertension and heart failure in hypertensive mice. Circulation. 2017;135(10):964–977. doi: 10.1161/CIRCULATIONAHA.116.024545. 27927713

[24] Kaye DM, Shihata W, Jama HA, Tsyganov K, Ziemann M, Kiriazis H, et al. Deficiency of prebiotic fibre and insufficient signalling through gut metabolite sensing receptors leads to cardiovascular disease. Circulation. 2020;0(0). 10.1161/CIRCULATIONAHA.119.043081 32093510

[25] Jama HA, Rhys-Jones D, Nakai M, Yao CK, Climie RE, Sata Y, Anderson D, Creek DJ, Head GA, Kaye DM, et al. Prebiotic intervention with HAMSAB in untreated essential hypertensive patients assessed in a phase II randomized trial. Nat Cardiovasc Res. 2023;2(1):35–43. doi: 10.1038/s44161-022-00197-4. 39196205

[26] R Muralitharan R, Nakai ME, Snelson M, Zheng T, Dinakis E, Xie L, Jama H, Paterson M, Shihata W, Wassef F, et al. Influence of angiotensin II on the gut microbiome: modest effects in comparison to experimental factors. Cardiovasc Res. 2024;120:1155–1163. doi: 10.1093/cvr/cvae062. 38518247 PMC11368123

[27] Desai MS, Seekatz AM, Koropatkin NM, Kamada N, Hickey CA, Wolter M, Pudlo NA, Kitamoto S, Terrapon N, Muller A, et al. A dietary fiber-deprived gut microbiota degrades the colonic mucus barrier and enhances pathogen susceptibility. Cell. 2016;167(5):1339–1353 e21. doi: 10.1016/j.cell.2016.10.043. 27863247 PMC5131798

[28] Hutchison ER, Kasahara K, Zhang Q, Vivas EI, Cross T-WL, Rey FE. Dissecting the impact of dietary fiber type on atherosclerosis in mice colonized with different gut microbial communities. NPJ Biofilms Microbiomes. 2023;9(1):31. doi: 10.1038/s41522-023-00402-7. 37270570 PMC10239454

[29] Cantu-Jungles TM, Bulut N, Chambry E, Ruthes A, Iacomini M, Keshavarzian A, Johnson TA, Hamaker BR, Zambrano MM. Dietary fiber hierarchical specificity: the missing link for predictable and strong shifts in gut bacterial communities. mBio. 2021;12(3), 10.1128/mbio.01028-21. PMC8262931 34182773

[30] De Filippo C, Cavalieri D, Di Paola M, Ramazzotti M, Poullet JB, Massart S, Collini S, Pieraccini G, Lionetti P. Impact of diet in shaping gut microbiota revealed by a comparative study in children from Europe and rural Africa. Proc Natl Acad Sci. 2010;107(33):14691–14696. doi: 10.1073/pnas.1005963107. 20679230 PMC2930426

[31] Walker RL, Vlamakis H, Lee JWJ, Besse LA, Xanthakis V, Vasan RS, Shaw SY, Xavier RJ. Population study of the gut microbiome: associations with diet, lifestyle, and cardiometabolic disease. Genome Med. 2021;13(1):188. doi: 10.1186/s13073-021-01007-5. 34915914 PMC8680346

[32] Garcia-Mantrana I, Selma-Royo M, Alcantara C, Collado MC. Shifts on gut microbiota associated to Mediterranean diet adherence and specific dietary intakes on general adult population. Front Microbiol. 2018;9:890. doi: 10.3389/fmicb.2018.00890. 29867803 PMC5949328

[33] De Filippis F, Pellegrini N, Vannini L, Jeffery IB, La Storia A, Laghi L, Serrazanetti DI, Di Cagno R, Ferrocino I, Lazzi C, et al. High-level adherence to a Mediterranean diet beneficially impacts the gut microbiota and associated metabolome. Gut. 2016;65(11):1812–1821. doi: 10.1136/gutjnl-2015-309957. 26416813

[34] Choo JM, Rogers GB. Gut microbiota transplantation for colonization of germ-free mice. STAR Protocols. 2021;2(3):100610. doi: 10.1016/j.xpro.2021.100610. 34189475 PMC8220245

[35] Sommer F, Bäckhed F. The gut microbiota — masters of host development and physiology. Nat Rev Microbiol. 2013;11(4):227–238. doi: 10.1038/nrmicro2974. 23435359

[36] Nicholson JK, Holmes E, Kinross J, Burcelin R, Gibson G, Jia W, Pettersson S. Host-gut microbiota metabolic interactions. Science. 2012;336(6086):1262–1267. doi: 10.1126/science.1223813. 22674330

[37] Luckey T. Germfree life and gnotobiology. Elsevier; 2012.

[38] Dang H, Feng P, Zhang S, Peng L, Xing S, Li Y, Wen X, Zhou L, Goswami S, Xiao M, et al. Maternal gut microbiota influence stem cell function in offspring. Cell Stem Cell. 2025;32(2):246–262 e8. doi: 10.1016/j.stem.2024.10.003. 39667939

[39] Wostmann B, Bruckner-Kardoss E. Development of cecal distention in germ-free baby rats. Am J Physiol Leg Content. 1959;197(6):1345–1346. doi: 10.1152/ajplegacy.1959.197.6.1345. 13846013

[40] Djurickovic SM, Ediger RD, Hong CC. Volvulus at the ileocaecal junction in germfree mice. Lab Anim. 1978;12(4):219–220. doi: 10.1258/002367778781088585. 732263

[41] Courtney CL. Cecal torsion in rodents associated with chronic administration of clinafloxacin. Toxicol Pathol. 2000;28(5):643–648. doi: 10.1177/019262330002800502. 11026598

[42] Bolsega S, Smoczek A, Meng C, Kleigrewe K, Scheele T, Meller S, Glage S, Volk H, Bleich A, Basic M. The genetic background is shaping cecal enlargement in the absence of intestinal microbiota. Nutrients. 2023;15(3):636. doi: 10.3390/nu15030636. 36771343 PMC9921660

[43] Macpherson AJ, de Agüero MG, Ganal-Vonarburg SC. How nutrition and the maternal microbiota shape the neonatal immune system. Nat Rev Immunol. 2017;17(8):508–517. doi: 10.1038/nri.2017.58. 28604736

[44] Ganal-Vonarburg SC, Hornef MW, Macpherson AJ. Microbial-host molecular exchange and its functional consequences in early mammalian life. Science. 2020;368(6491):604–607. doi: 10.1126/science.aba0478. 32381716

[45] Smith K, McCoy KD, Macpherson AJ. Use of axenic animals in studying the adaptation of mammals to their commensal intestinal microbiota. Semin Immunol. 2007;19(2):59–69. doi: 10.1016/j.smim.2006.10.002. 17118672

